# A Midpoint Process Evaluation of the Los Angeles Basin Racial and Ethnic Approaches to Community Health Across the US (REACH US) Disparities Center, 2007-2009

**Published:** 2011-08-15

**Authors:** Annette E. Maxwell, Antronette K. Yancey, Mona AuYoung, Joyce J. Guinyard, Beth A. Glenn, Ritesh Mistry, Paul A. Simon, Roshan Bastani, William J. McCarthy, Jonathan E. Fielding

**Affiliations:** UCLA Department of Cancer Prevention and Control Research, School of Public Health and Jonsson Comprehensive Cancer Center; University of California, Los Angeles (UCLA), and Jonsson Comprehensive Cancer Center, Los Angeles, California; University of California, Los Angeles (UCLA), and Jonsson Comprehensive Cancer Center, Los Angeles, California; University of California, Los Angeles (UCLA), and Jonsson Comprehensive Cancer Center, Los Angeles, California; University of California, Los Angeles (UCLA), and Jonsson Comprehensive Cancer Center, Los Angeles, California; University of California, Los Angeles (UCLA), and Jonsson Comprehensive Cancer Center, Los Angeles, California; University of California, Los Angeles (UCLA), and Jonsson Comprehensive Cancer Center, Los Angeles, California; University of California, Los Angeles (UCLA), and Jonsson Comprehensive Cancer Center, Los Angeles, California; University of California, Los Angeles (UCLA), and Jonsson Comprehensive Cancer Center, Los Angeles, California; UCLA and Jonsson Comprehensive Cancer Center, Los Angeles County Department of Public Health, Los Angeles, California

## Abstract

**Background:**

Racial/ethnic minority groups have higher risks for disease resulting from obesity.

**Community Context:**

The University of California, Los Angeles, and the Los Angeles County Department of Public Health partnered with community organizations to disseminate culturally targeted physical activity and nutrition-based interventions in worksites.

**Methods:**

We conducted community dialogues with people from 59 government and nonprofit health and social service agencies to develop wellness strategies for implementation in worksites. Strategies included structured group exercise breaks and serving healthy refreshments at organizational functions. During the first 2 years, we subcontracted with 6 community-based organizations (primary partners) who disseminated these wellness strategies to 29 organizations within their own professional networks (secondary worksites) through peer modeling and social support. We analyzed data from the first 2 years of the project to evaluate our dissemination approach.

**Outcome:**

Primary partners had difficulty recruiting organizations in their professional network as secondary partners to adopt wellness strategies. Within their own organizations, primary partners reported significant increases in implementation in 2 of the 6 core organizational strategies for promoting physical activity and healthy eating. Twelve secondary worksites that completed organizational assessments on 2 occasions reported significant increases in implementation in 4 of the 6 core organizational strategies.

**Interpretation:**

Dissemination of organizational wellness strategies by trained community organizations through their existing networks (train-the-trainer) was only marginally successful. Therefore, we discontinued this dissemination approach and focused on recruiting leaders of organizational networks.

## Introduction

More than one-third of adults and nearly one-fifth of children in the United States are obese, placing them at greater risk for heart disease, diabetes, and other chronic diseases (1,2). People of racial/ethnic minority groups experience a higher disease burden. For example, heart disease deaths among African Americans younger than 65 years (31%) is twice that of whites (15%) (3). Latinos have high rates of obesity, a major contributor to heart disease and stroke, and are increasingly likely to adopt unhealthy diets as they acculturate (4). Asians may experience chronic disease comorbidities even though they are not obese (5,6). Reasons underlying obesity-related disparities include socioeconomic, physical, and social environmental influences (eg, marketing, neighborhood characteristics, social norms, discrimination) (7).

## Community Context

The Centers for Disease Control and Prevention (CDC) created Racial and Ethnic Approaches to Community Health Across the US (REACH US) (8), a national initiative to combat health disparities. REACH US supports 40 grantee partners that establish community-based programs and culturally appropriate interventions to eliminate health disparities. One of these CDC-funded partners, the Los Angeles Basin Center of Excellence in the Elimination of Health Disparities (CEED), is a collaboration of the University of California, Los Angeles (UCLA), the Los Angeles County Department of Public Health, and partnering community-based health and social service organizations. On the basis of prior work and community input (9-12), CEED decided to focus on the primary prevention of heart disease, stroke, and cancer through culturally targeted promotion of physical activity and healthy nutrition via organizational change.

Intervening at key community institutions such as worksites, churches, and schools can have a profound effect on obesity prevention, because these institutions represent contexts for engaging captive audiences to make changes in social and cultural norms, enhance awareness, build necessary skills, and make structural shifts to support desired behavior change (13). Employees spend a substantial portion of their lives in these settings. The importance of intervening in such settings is heightened for low-income populations, who generally live in obesogenic environments and have little free time and few other resources to devote to physical activity and healthy nutrition. Furthermore, norm changes initiated in these community institutions may transfer to other settings. For example, clinic workers may influence client behaviors (14) and serve as agents of positive change within their own families and social circles. The Task Force on Community Preventive Services has recommended worksite interventions that combine healthy nutrition and promotion of physical activity (15).

We describe our approach to disseminating wellness strategies at worksites in Los Angeles and Orange counties from 2007 to 2009, the first 2 years of CEED. We present results of a midpoint process evaluation that reshaped our approach to dissemination during the next 2 years.

## Methods

### Selection of wellness strategies

With the support of a National Institutes of Health disparities grant, and before we received CEED funding, we conducted a series of community dialogues with 188 representatives of 59 different government and nonprofit health and social service organizations. We presented wellness strategies that were practice- or evidence-based to these representatives, who then identified those strategies that could realistically be implemented in their organizations during work hours (16). The 6 core organizational strategies arising from these dialogues are holding 10-minute structured group activity (the Instant Recess program [16]) breaks; having walking meetings; providing healthy refreshments at organizational meetings and functions; replacing desktop candy dishes with fruit baskets; offering healthy, competitively priced foods in vending machines, cafeterias, and from onsite food vendors; and promoting stair use by posting prompts and improving stair accessibility, visibility, and appeal. Because worksite wellness efforts have been most focused on and successful at nutrition change, we concentrated on physical activity, particularly "push" or "opt-out" strategies that made the active choice the default choice. These strategies were culturally targeted by integrating movement to music in group settings as a routine part of daily work, which is congruent culturally for women and racial/ethnic minority populations; grounding physical activity moves in culturally relevant sports and dance traditions with appropriate music; and incorporating traditional foods of various cultures in healthy nutrition suggestions (eg, fruit selected for fruit baskets, vegetable or legume dishes in recipes for potlucks).

### Dissemination of wellness strategies

In the first 2 years of CEED, we subcontracted with 6 community-based health and social service organizations as primary partners, organizations with a history of successful outreach activities in the target communities. Given the racial and ethnic diversity of Los Angeles County, we partnered with organizations that served the largest local minority populations, including Latinos, African Americans, and Asians and Pacific Islanders. For this discussion, we refer to these partners as Agencies A through F. Agency A is a community health center that provides services to low-income families, particularly Asians and Pacific Islanders. Agency B empowers black women to take responsibility for their health and to advocate for changes in policies that adversely affect their health. Agency C, located in a majority-Latino neighborhood, develops and preserves affordable housing; advocates for childcare, quality education, and health care access; and promotes economic development and progressive public policy. Agency D trains Latino community health workers to be leaders in fostering wellness through provision of quality preventive services and educational programs. Agency E enables African Americans and other racial/ethnic minority groups to attain economic self-reliance, parity, power, and civil rights through advocacy activities and the provision of programs and services. Agency F addresses the needs of underserved, predominantly Latino, families by providing information at worksites that encourages healthy lifestyles and timely and appropriate use of health care services.

The number of employees at each organization ranges from fewer than 10 to approximately 300. The racial/ethnic composition of the employees reflects that of the agency's clientele. Five of the community partner organizations are in Los Angeles County and 1 is in Orange County. One of the organizations has been in existence for 85 years, and all others, between 15 and 25 years.

Primary partners received financial support and technical assistance to implement selected policy and practice changes within their own organizations and to participate in the worksite wellness assessments associated with CEED. Each primary partner also agreed to recruit and train 5 to 13 organizations (secondary partners), depending on their capacity, from their network of collaborators willing to implement selected policy and practice changes. The goal of this "train-the-trainer" model was to disseminate the 6 core organizational strategies to the larger community through diverse organizations offering peer modeling and social support. The director of each primary partner agency and the principal investigator at UCLA (A.Y.) signed a memorandum of understanding that outlined the scope of work.

UCLA staff provided technical assistance to all primary sites in person and by telephone and e-mail, including at least 2 site visits per year. Other trainings and events were made available for both primary and secondary partners, including Instant Recess trainings on how to lead and implement regular structured group activity breaks (a total of 5 trainings during the first 2 years), 1 workshop on use of evidence-based strategies, the California REACH conference, and an annual community cancer prevention symposium. In addition, designated peer leaders at each worksite attended the Program Champion training, a program designed to build skills in advancing fitness-promoting practices and policies at the workplace.

### Coalition advisory board

A coalition advisory board comprising members from the Los Angeles County Department of Public Health, academia, and 10 community agencies was convened in the first year. The advisory board meets quarterly. Its goal is to provide oversight to CEED to assure cultural relevance, appropriateness, and responsiveness of the interventions; to support dissemination efforts; and to ensure the establishment of an authentic partnership between the community partners, UCLA, and the Los Angeles County Department of Public Health. The advisory board chose its own name (Cultivating Healthy Activities Together), identified its guiding principles, and established ground rules for meetings. Members determined that 4 central elements of community-based participatory research would shape the coalition: partnership, participation, equity, and social change.

### Assessment tools

We asked staff at each community organization to complete a worksite wellness assessment (WWA) to identify current practice and policy support for health promotion activities before initiating any CEED activities (baseline) and again at 6 and 12 months after the organization's enrollment. The assessment tool was adapted for an earlier REACH project from the New York State Department of Health Heart Check, a validated instrument assessing organizational characteristics that support heart-healthy behaviors (nutrition, physical activity, smoking cessation, stress reduction, screening) with demonstrated sensitivity in detecting preintervention and postintervention changes (12,17-19). The adaptation process, consisting primarily of adding physical activity policy and practice items, was described in a previous article (12). The WWA assesses worksite policy and environmental factors that promote physical activity (eg, conducting activity breaks during work hours) and healthy food choices (eg having nutrient-rich foods in vending machines).

Each site was asked to return 3 WWAs, to be completed by 1 representative each from upper management, middle management, and nonmanagement or line staff. We chose different levels of the organizational hierarchy to capture and compare the range of perspectives of both the decision makers and line staff. Initially, the WWA comprised a 13-page self-administered questionnaire for line staff and a 15-page questionnaire for upper management. During the first assessment period (2007 to 2008) we noticed that many assessments were only partially completed, and our community partners thought that the instruments were too long and cumbersome. Subsequently, we shortened the assessment instrument to 6 pages with the same instrument to be completed by line staff and management, and we made it available online.

UCLA staff also conducted 1 environmental audit at each primary partner organization to verify self-reported implementation of wellness strategies. Specifically, staff observed and noted the availability of drinking water and fruit baskets in common areas, contents of vending machines, and incidence of physical activity breaks during work routine. During these site visits, UCLA staff also provided technical assistance as needed.

### Statistical analysis

Data from the WWAs were entered into a database in EpiData version 3.1 (EpiData Corporation, Odense, Denmark) and then exported to Stata version 10.0 (StataCorp LP, College Station, Texas) for analysis. Sites varied in how many respondents completed the organization-level questionnaire. Although most WWAs were completed by the same employees at baseline and follow-up, some were completed by different employees. Prevalence estimates were obtained by using Stata's survey data cross-tabulation procedure. For the comparison of baseline and follow-up data, we combined formal and informal policy changes, combined missing values with the "no" categories, and weighted the data based on the number of responses received from each organization. Thus, all worksites made an equal contribution to overall estimates of the proportion of worksites supporting specific nutrition and physical activity-related policies and practices. The significance of the difference in mean prevalence estimates between baseline and 8-month follow-up was obtained by using Stata's survey data mean procedure and Stata's postestimation Wald test to determine whether the follow-up mean was equal to the baseline mean prevalence estimate. CEED was ruled exempt from UCLA Human Subjects Protection Committee review because interventions and assessments were focused at the organizational level, not at the individual level, and UCLA's role was one of training and consultation, not direct-service delivery.

## Outcome

### Recruiting worksites through primary community partners

The 6 primary community partner organizations agreed to recruit 41 to 53 secondary worksites during the first 2 years (Table 1). However, only 29 of the 99 secondary worksites (29%) that were contacted actually enrolled by completing at least the baseline assessment for their own worksites. Because of the lower-than-expected participation rate, primary organizations had to contact more secondary organizations than was planned. Overall, participating organizations were distributed over an area of approximately 418 square miles in Los Angeles and Orange counties. Primary sites reported the following barriers to recruitment: need to focus on current priorities rather than on creating new programs because of budget cuts and staff layoffs or turnover; inability to focus on wellness; inability to secure commitment from upper management, even with multiple lower levels in the organizational hierarchy expressing interest or enthusiasm in wellness; employee safety concerns regarding risks of injury during exercise breaks (expressed primarily by human resources personnel); and lack of financial compensation for secondary sites. In addition, by year 2 of the project, primary sites had reached a saturation point in their spheres of influence, having already approached many of the organizations in their professional network. They were less equipped to identify new sites to recruit, lowering the yield, with only 12 organizations completing at least 1 worksite wellness assessment in year 2 compared with 17 in year 1.

### Quality of worksite wellness assessments

We compared the answers to 3 questions from the WWAs completed by members of the primary community partner organizations with answers to the same questions on the 8-month follow-up questionnaire and to findings from the environmental audits (Table 2). At both baseline and follow-up, several sites submitted more than the 3 completed surveys that we expected. The presence of functional water fountains, taps, or water coolers at worksites was reported consistently, and audits confirmed this information. The reporting on the presence of fruit bowls and the conduct of exercise breaks during work hours was less consistent and did not always agree with audit observations. However, organizations may have conducted exercise breaks at times other than when the audits were performed.

### Implementation of worksite wellness strategies

Summary data from the WWAs for primary sites (Table 3) show that at baseline, the extent to which core strategies were implemented ranged from 31% (having policies regarding healthy food procurement, weighted value) to 98% (having functional water coolers, weighted value). In addition, policies regarding nutrient-rich food and beverages and food procurement were mostly described as informal rather than formal or data were missing.

For the 6 primary worksites, there were fewer missing data at follow-up than at baseline. This may be due both to more diligent completion of questionnaires and better quality control by UCLA staff. The proportion of organizations who reported "healthier" trends between baseline and follow-up significantly increased for policies regarding healthy food procurement and exercise breaks conducted during work hours. There were no changes with respect to policies regarding nutritious food and beverages at company meetings, the presence of functional water coolers, and the support of standing, stretching, or fidgeting during meetings. Decreased support reported for casual dress attire during work hours was significant.

Similar trends were observed in secondary sites (Table 4). Support of policies for nutritious food and beverages increased significantly from baseline to follow-up as did exercise breaks conducted during work hours. In addition, there was an increase in availability of functional water coolers or fountains, from 83% at baseline to 99% at follow-up (weighted values). Support decreased for casual dress attire and for standing, stretching, or fidgeting during meetings. It should be noted that only 12 out of 29 secondary sites completed follow-up WWAs, and differences between implementation of these core strategies among those secondary sites that completed WWAs at baseline and those that did not (data not shown) were significant. However, on the basis of the 6 core strategies that we assessed at baseline, we detected no systematic drop-out bias.

More primary sites reported exercise breaks during work hours at baseline than did secondary sites. This may be explained by the participation of 2 of the primary sites in a previous pilot study that promoted exercise breaks during work hours, attesting to the sustainability of this approach given adequate implementation support (16).

## Interpretation

On the basis of the findings of these process measures, we shortened the WWA and modified the protocol to have the same employee (a senior-level but not director-level manager) complete baseline and follow-up assessments in future years. Informed by the data presented in this report and discussions with our community partners, we decided to discontinue the dissemination strategy employed from 2007 to 2009. Instead of the projected participation of 41 to 53 secondary sites, we enrolled only 29 sites, which completed at least a baseline WWA. In addition, primary sites reported that it took a lot more effort than anticipated to recruit secondary sites. Furthermore, only 12 of the secondary sites completed a follow-up assessment. The 17 sites that did not complete WWAs at follow-up likely did not implement the recommended wellness strategies.

Our data do suggest, however, that participating sites were able to incorporate several of the core strategies into worksite routines, especially exercise breaks. According to Luanne Heinen, National Business Group on Health (personal communication June 15, 2010), this is noteworthy in that the typical corporate "pull" physical activity promotion strategies that rely on individual motivation (eg, onsite fitness centers, gym membership subsidies) are being abandoned as costly and ineffective by many corporations (20). There was also a significant increase in the adoption of policies (albeit mainly informal) promoting nutritious food and beverages. We will encourage our community partners to formalize these policies to ensure sustainability.

The principal investigator (A.Y.) and staff had actively promoted the centerpiece wellness strategy, the 10-minute Instant Recess break, for the past decade by using accelerated funding from the CEED. They have offered workshops, mounted a website, and led recess breaks at many local and national meetings and at any type of gathering that involved sitting for long periods. As a result, many community members and leaders, researchers, and public health practitioners participated in various activity breaks that were built into the agendas of these meetings. These breaks are generally welcomed as an opportunity to relieve stress and restore energy. We have, over time, received increasing numbers of requests from organizations, networks, and public agencies for trainings, materials, and related resources to implement recess breaks. Working with these groups led to the development and adoption of a new dissemination model, the Meta-Volition Model, in which "sparkplugs" (public health leaders actively promoting fitness) engage self-identified early-adopter leaders who are connected to networks of organizations to encourage them to implement "push" organizational wellness policy and practice changes (20). On the basis of some of the findings of our midpoint evaluation, we are increasingly using the Meta-Volition Model in an attempt to extend and expand the visibility, reach, and exposure of the policies and practices promoted. We will evaluate this new dissemination approach during the remaining years of the project.

## Figures and Tables

**Table 1:** Recruitment of Secondary Community Sites by Primary Community Partner Agencies, Los Angeles Basin Center of Excellence in the Elimination of Health Disparities, 2007-2009

**Primary Community Partner Agency/Fiscal Year**	No. of Planned Secondary Sites to Be Recruited	No. of Secondary Sites Contacted^a^	No. of Secondary Sites Participating^b^	% of Secondary Sites Contacted That Participated
**A**				
2007-2008	3-5	10	2	20
2008-2009	3	6	1	17
Total	6-8	16	3	19
**B**				
2007-2008	4-6	4	3	75
2008-2009	3	8	2	25
Total	7-9	12	5	42
**C**				
2007-2008	3-5	7	1	14
2008-2009	2	2	2	100
Total	5-7	9	3	33
**D**				
2007-2008	4-6	14	3	21
2008-2009	3	10	4	40
Total	7-9	24	7	29
**E**				
2007-2008	3-4	3	1	33
2008-2009	3	7	3	43
Total	6-7	10	4	40
**F**				
2007-2008	5-7	21	7	33
2008-2009	5-6	7	0	0
Total	10-13	28	7	25
**Subtotal year 1, 2007-2008**	22-33	59	17	29
**Subtotal year 2, 2008-2009**	19-20	40	12	30
**Total, 2007-2009**	41-53	99	29	29

a More secondary sites were contacted than planned because of the lower-than-expected participation rate.

b Completed at least 1 worksite wellness assessment.

**Table 2:** Comparison of Responses to 3 Questions on WWA and Environmental Audit, By Primary Community Partner Agency, Los Angeles Basin Center of Excellence in the Elimination of Health Disparities, 2007-2009

**Question/Primary Partner Agency**	Baseline WWA Response	8-Month WWA Response^a^	Environmental Audit^b^
**Are there functional drinking fountains, taps, or water coolers present within the worksite?**			
A	3 yes, 0 no	4 yes, 0 no	Yes
B	3 yes, 0 no	3 yes, 0 no	Yes
C	3 yes, 0 no	6 yes, 1 no	Yes
D	10 yes, 0 no	7 yes, 0 no	Yes
E	3 yes, 0 no	3 yes, 0 no	Yes
F	4 yes, 0 no	3 yes, 0 no	Yes
**Does your worksite provide a bowl of fresh fruit in the reception or central area?**			
A	1 yes, 1 no	1 yes, 3 no	No
B^c^	NC	0 yes, 3 no	No
C^c^	NC	1 yes, 6 no	Yes
D	1 yes, 0 no	6 yes, 1 no	Yes
E^c^	NC	0 yes, 3 no	No
F	0 yes, 1 no	3 yes, 0 no	Yes
**Are exercise breaks conducted during meetings or at predesignated times of the workday?^d^**			
A	0 yes, 3 no	0 yes, 4 no	No
B	1 yes, 2 no	3 yes, 0 no	No
C	1 yes, 0 no	2 yes, 4 no	No
D	4 yes, 6 no	5 yes, 2 no	No
E	1 yes, 2 no	2 yes, 1 no	No
F	3 yes, 1 no	2 yes, 0 no	No

Abbreviations: WWA, worksite wellness assessment; NC, not calculated.

a WWAs were completed 7 to 10 months after baseline, with an average of 8 months.

b Audits were conducted at the same time as the follow-up WWA ± 2 months, except for organization C.

c Responses were missing on the baseline questionnaire.

d Exercise breaks not observed during the audit may have been held at another time.

**Table 3:** Responses (n = 27) to WWA at 6 Primary Community Partner Agencies, Los Angeles Basin Center of Excellence in the Elimination of Health Disparities, 2007-2009

WWA Question/Response	Unweighted		Weighted^a^		% Change (P Value)^b^

	Baseline, n (%)	Follow-Up, n (%)	Baseline%	Follow-Up %	
**Policies regarding nutritious food and beverages at company meetings**					
Informal	12 (44)	14 (52)	60	73	+22 (.14)
Formal	4 (15)	4 (15)			
None	4 (15)	5 (19)	40	27	NC
Missing	7 (26)	4 (15)			
**Policies regarding healthy food procurement**					
Informal	5 (19)	13 (48)	31	61	+97 (<.001)
Formal	2 (7)	2 (7)			
None	3 (11)	10 (37)	69	39	NC
Missing	17 (63)	2 (7)			
**Functional water coolers or fountain**					
Yes	26 (96)	26 (96)	98	98	0 (.87)
No	0 (0)	1 (4)	2	2	NC
Missing	1 (4)	0 (0)			
**Casual dress attire supported during work hours**					
Yes	15 (56)	10 (37)	60	39	−35 (.04)
No	11 (41)	12 (44)	40	61	NC
Missing	1 (4)	5 (19)			
**Standing, stretching, or fidgeting supported during meetings**					
Yes	18 (67)	17 (63)	72	67	−7 (.56)
No	7 (26)	8 (30)	28	33	NC
Missing	2 (7)	2 (7)			
**Exercise breaks conducted during meetings or predesignated times of work day**					
Yes	10 (37)	17 (63)	35	56	+60 (.01)
No	14 (52)	8 (30)	65	46	NC
Missing	3 (11)	2 (7)			

Abbreviation: WWA, worksite wellness assessment; NC, not calculated.

a For analyses, data were weighted, and response categories were dichotomized as "% yes" (informal and formal) and "% no" (none, no, and missing).

b Percentage change of desirable behavior from baseline to follow-up (Wald test).

**Table 4:** Responses (N = 42) to WWA at 12 Secondary Community Partner Agencies, Los Angeles Basin Center of Excellence in the Elimination of Health Disparities, 2007-2009

WWA Question/Response^a^	Unweighted		Weighted^a^		% Change^b^ (P value)
	Baseline, n (%)	Follow-Up, n (%)	Baseline, %	Follow-Up, %	
**Policies regarding nutritious food and beverages at company meetings**					
Informal	18 (43)	27 (64)	54	81	+50(<.001)
Formal	6 (14)	7 (17)			
None	18 (43)	8 (19)	46	19	NC
Missing	0 (0)	0 (0)			
**Policies regarding healthy food procurement**					
Informal	3 (7)	18 (43)	7	53	+657(<.001)
Formal	0 (0)	4 (10)			
None	8 (19)	14 (33)	93	47	NC
Missing	31 (74)	6 (14)			
**Functional water coolers or fountain**					
Yes	36 (86)	41 (98)	83	99	+19(<.001)
No	6 (14)	1 (1)	17	1	NC
Missing	0 (0)	0 (0)			
**Casual dress attire supported during work hours**					
Yes	32 (76)	28 (67)	76	64	−16(<.01)
No	10 (24)	13 (31)	24	36	NC
Missing	0 (0)	1 (2)			
**Standing, stretching, or fidgeting supported during meetings**					
Yes	16 (38)	13 (31)	42	32	−24(.07)
No	25 (60)	29 (69)	58	68	NC
Missing	1 (2)	0 (0)			
**Exercise breaks conducted during meetings or predesignated times of work day**					
Yes	2 (5)	21 (50)	8	50	+525(<.001)
No	40 (95)	18 (43)	92	50	NC
Missing	0 (0)	3 (7)			

Abbreviations: WWA, Worksite Wellness Assessment; NC, not calculated.

a For analyses, data were weighted, and response categories were dichotomized as "% yes" (informal and formal) and "% no" (none, no, and missing).

b Percentage change of desirable behavior from baseline to follow-up (Wald test).
